# The k-junction motif in RNA structure

**DOI:** 10.1093/nar/gku144

**Published:** 2014-02-14

**Authors:** Jia Wang, Peter Daldrop, Lin Huang, David M. J. Lilley

**Affiliations:** Cancer Research UK Nucleic Acid Structure Research Group, MSI/WTB Complex, The University of Dundee, Dow Street, Dundee DD1 5EH, UK

## Abstract

The k-junction is a structural motif in RNA comprising a three-way helical junction based upon kink turn (k-turn) architecture. A computer program written to examine relative helical orientation identified the three-way junction of the *Arabidopsis* TPP riboswitch as an elaborated k-turn. The *Escherichia coli* TPP riboswitch contains a related k-junction, and analysis of >11 000 sequences shows that the structure is common to these riboswitches. The k-junction exhibits all the key features of an N1-class k-turn, including the standard cross-strand hydrogen bonds. The third helix of the junction is coaxially aligned with the C (canonical) helix, while the k-turn loop forms the turn into the NC (non-canonical) helix. Analysis of ligand binding by ITC and global folding by gel electrophoresis demonstrates the importance of the k-turn nucleotides. Clearly the basic elements of k-turn structure are structurally well suited to generate a three-way helical junction, retaining all the key features and interactions of the k-turn.

## INTRODUCTION

The kink turn (k-turn) is an extremely widespread structural motif that generates a tight kink in duplex RNA (1,2), thereby frequently mediating tertiary interactions. This is exploited by at least six riboswitch structures to create ligand binding pockets, and there are numerous k-turn structures found in ribosomal RNA species contributing to the architecture of the ribosome (1). Many k-turns are also targets for the binding of specific proteins, including the L7Ae family (3). For example, the assembly of the box C/D and H/ACA snoRNPs is initiated by the binding of an L7Ae protein to a k-turn (4–6). The kinked structure of the k-turn requires stabilization, in the absence of which the RNA is relatively extended and probably flexible. K-turn stabilization can occur due to the presence of metal ions for some (but not all) sequences (7), as a result of tertiary interactions (8) or due to the binding of proteins (9–12).

**Figure 1.**
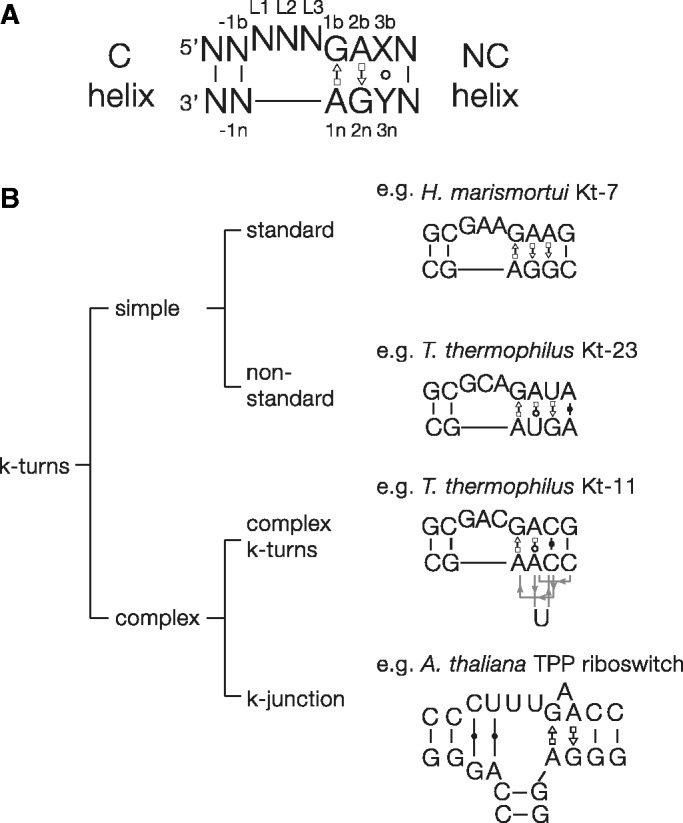
K-turn sequences and classification. (**A**) The secondary structure of a simple, standard k-turn. Our standard nomenclature is used to designate nucleotide positions. The 3b•3n pair is frequently non-Watson–Crick. (**B**) A classification of k-turn structures. The k-turns can be divided into simple and complex. Examples are shown for each class. Simple k-turns are further divided into standard and non-standard, with the critical G•A pairs preserved or substituted, respectively. The complex k-turns exhibit a greater departure from the standard k-turn, where the primary sequence does not map onto the 3D RNA structure in a linear way. We now may consider the k-junctions discussed here as another branch of the complex k-turns.

The standard k-turn comprises duplex RNA with a three-nucleotide bulge followed by G•A and A•G pairs (Figure 1). The nucleotides are named according to a universal scheme (13). In the folded k-turn, the 5′-nucleotide of the loop (L1) is stacked onto the end of the C helix, L2 is stacked onto the end of the NC helix, while L3 is directed away from the k-turn into the solvent. The folded structure is stabilized by a number of H-bonding interactions within the core (10,13–15). Two cross-strand H-bonds are conserved and critical. These are donated by the O2′ atoms of L1 (13) and –1n (15) to the conserved adenine nucleobases 1n and 2b, respectively. The latter can be accepted either by A2b N3 or N1, dividing the known k-turn structures into the N3 and N1 class k-turns (15).

The k-turns can be classified into different groups based on sequence and structure (Figure 1). The simple k-turn is a double-stranded RNA with a bulge that is followed by the A•G pairs of the NC helix. These can be subdivided into standard and non-standard simple k-turns. The standard simple k-turn has G•A and A•G pairs at the 1b•1n and 2b•2n positions respectively, exemplified by *Haloarcula marismortui* Kt-7 or the human U4 snRNA k-turn. Non-standard simple k-turns have a substitution in one of the G•A pairs. For example, in Kt-23 sequences of 30S ribosomal subunits of different species the 2n position has a frequency U>C>G>A, although examples analysed can form normal k-turn structures despite the departure from the standard sequence (16,17).

In the complex k-turns the nucleotides contributing to the G•A pairs do not map linearly onto the sequence of the RNA, although the structure formed is recognizably a normal k-turn. Applying our k-turn nomenclature (13), we identify nucleotides according to their position in the 3D structure, rather than in the primary sequence. In *Thermus thermophilus* Kt-11 the non-bulged strand of the NC helix doubles back on itself to form an S-turn, such that at the level of the primary sequence the 1n and 2n nucleotides are separated by two nucleotides including the cytosine at the 3n position (Figure 1). Nevertheless, the A2b is placed normally within the structure so that it accepts a hydrogen bond from –1n O2′ to form an N1 class k-turn. In Kt-15 of *H.marismortui* the adenine that approximates to the 2b position is actually contributed by the non-bulged strand, and a triple G2n•U•A2b interaction is formed. Yet the structure is still basically a k-turn, with a normal G1b•A1n base pair and the usual L1 O2′ to A1n N1 hydrogen bond. Indeed Kt-15 is the natural ribosomal binding site for the L7Ae protein.

The complex k-turns show that the sequence of the motif can be significantly altered from the standard form, yet fold to retain all the key features of the k-turn in three dimensions. These structures would be difficult to detect from an analysis of sequence alone. We thus considered whether the fundamental elements of k-turn structure might exist within more complex structures, and perhaps have escaped detection. We therefore wrote a computer program that would search within RNA structures for two helices with a relative inclination that was similar to that of the geometry of the C and NC helices of a standard k-turn. This analysis revealed that the thiamine pyrophosphate (TPP) riboswitches contain a hitherto unsuspected motif that is a k-turn that has been elaborated into a three-helix junction. This nevertheless retains all the key interactions found in a simple k-turn. We term this structure the k-junction, adding another branch to the scheme shown in Figure 1.

## MATERIALS AND METHODS

### Computational methods

RNA structures were analysed using software written in Python 2.7 together with the BIOPYTHON package. The scripts are available for download in Supplementary Materials. Visual analysis of structures and the computational results was performed in PyMOL 1.5.3. The software was implemented on a standard desktop workstation running Windows 7. RNA-containing PDB files were downloaded from the Protein Data Bank (pdb.org) and stored on the local hard drive. Non-RNA atoms were removed from these files and only files containing at least ten complete nucleotides were retained.

All standard Watson–Crick base pairs, including the G–U wobble pair were identified, and for each structure minimal helical segments consisting of two base pairs in close proximity and suitable relative orientation were identified, and abstracted to sets of four vectors. The first vector defines the location of the helix segment, while vectors 2–4 define the orientation of the helix segment and are orthonormal. The second vector defines the main helix axis, the third the direction of the Watson–Crick hydrogen bonds projected to the plane perpendicular to the main helix axis, and the fourth is defined by being orthonormal to the second and third vectors. Therefore, these vectors define a coordinate system, with its origin located at this particular base pair and its coordinate axes aligned with the main features of the helix segment.

The k-turn motif search pattern was defined by calculating the relative coordinates of helix segments from the C and NC helices of known k-turn structures. To do this, helix segments from the NC helix were expressed in terms of the coordinate system defined by a helix segment from the C-helix and the relative location and orientation was recorded. For each structure the identified helix segments were checked against pairs matching the relative location and orientation of helix segments from the search pattern. Positively scoring helix segments were written to output files. Helix segments from each were saved as residues consisting of pseudo-atoms into a PDB file. These structures were then analysed by visual inspection.

### TPP riboswitch sequence alignment and analysis

A full alignment of 11 197 TPP riboswitch sequences taken from the Rfam database (18), accession number RF00059 was made using the latest version of Jalview (Jalview 2.8). We manually aligned the k-junction region against the two available crystal structures. The atomic resolution crystal structures of *Arabidopsis thaliana* TPP riboswitch (3D2G) and *E. coli* TPP riboswitch (2DGI) were used to define secondary structure masks for the sequence alignments. All sequence composition and covariation analysis was calculated using a modified version of Jalview which was kindly provided by Dr James Procter (University of Dundee).

### Preparation of RNA

Transcription templates were prepared by PCR amplification from a plasmid-borne gene for the TPP riboswitch using primers hybridizing to the T7 promoter and the 3′-end of the riboswitch. RNA was transcribed from 75 μg/ml DNA template in 30 mM Tris–HCl (pH 8.0), 10 mM DTT, 0.1% Triton X-100, 0.1 mM spermidine–HCl, 4 mM each NTP (Sigma; pH adjusted to 8.0), 40 mM MgCl_2_, 50 μg/ml T7 RNA polymerase, 1 U/ml inorganic pyrophosphatase (Sigma), for 3 to 3.5 h at 37°C. The products were purified by gel electrophoresis under denaturing conditions. RNA was extracted from gel slices by electroelution and exchanged five times into 40 mM K-Hepes (pH 7.5), 100 mM KCl, 10 mM MgCl_2_ for ITC analysis, or 25 mM Tris, 192 mM glycine (pH 8.3), 1 mM MgCl_2_ for analysis by gel electrophoresis. Concentrations were measured by absorbance at 260 nm using extinction coefficients calculated from the nucleotide composition and a hypochromic effect correction factor.

The sequence (written 5′ to 3′) of the unmodified *E. coli* TPP riboswitch was:

GGACUCGGGGUGCCCUUCUGCGUGAAGGCUGAGAAAUACCCGUAUCACCUGAUCUGGAUAAUGCCAGCGUAGGGAAGUUC

### Isothermal titration calorimetry

Microcalorimetric measurements of TPP binding to the *E. coli* TPP riboswitch and variants were performed by isothermal titration calorimetry (ITC) at 303.15 K as described by Kulshina *et al.*(19). The sequence of the TPP riboswitch was that written above together with substitutions noted in the text. Calorimetric data were fitted to a single-site binding model, where possible, using MicroCal ORIGIN software. Individual heat changes Δ*Q* at constant pressure are given by:
(1)
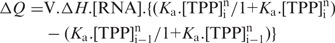

where Δ*H* is the change in enthalpy, V is the reaction volume, *K*_a_ is the association constant for TPP binding, and [TPP]*_i_* is the TPP concentration at the *i-*th injection.

### Gel electrophoretic analysis of folding

The electrophoretic mobility of TPP riboswitch RNA and sequence variants was analysed in 10% polyacrylamide gels in 25 mM Tris, 192 mM glycine (pH 8.3), 1 mM MgCl_2_ with or without the addition of TPP at the stated concentration at 25°C. RNA was preincubated in 25 mM Tris, 192 mM glycine (pH 8.3), 1 mM MgCl_2_ with or without TPP for 5 min at 20°C before addition of glycerol to 25% and loading onto the gel. Electrophoresis was performed either at 4 W for 2 h or 3 W for 2.5 h without or with (respectively) TPP added to the running buffer, such that the temperature remained below 20°C. The contents of anode and cathode chambers were mixed to prevent depletion of Mg^2+^ ions. After electrophoresis RNA was visualized by UV shadowing.

## RESULTS

### Search for k-turn-like structures

We wrote a computer program in Python 2.7 to analyse relative orientation of segments of double helix in coordinate files of RNA structures downloaded from the PDB. A search pattern was defined by calculating the relative coordinates of helix segments from the C and NC helix of known k-turn structures. This structural criterion was then applied to other RNA structures to search for sections in which two helical segments had a relative orientation that was similar to those of a conventional k-turn. For all helix segments we searched for nearby candidate helical segments that matched the search pattern. Using this approach we observed that the *A. thaliana* TPP riboswitch (PDB code 3D2G) contained two helices with a strong structural similarity to the k-turn geometry.

### The three-helix junction of the *A. thaliana* TPP riboswitch

**Figure 2.**
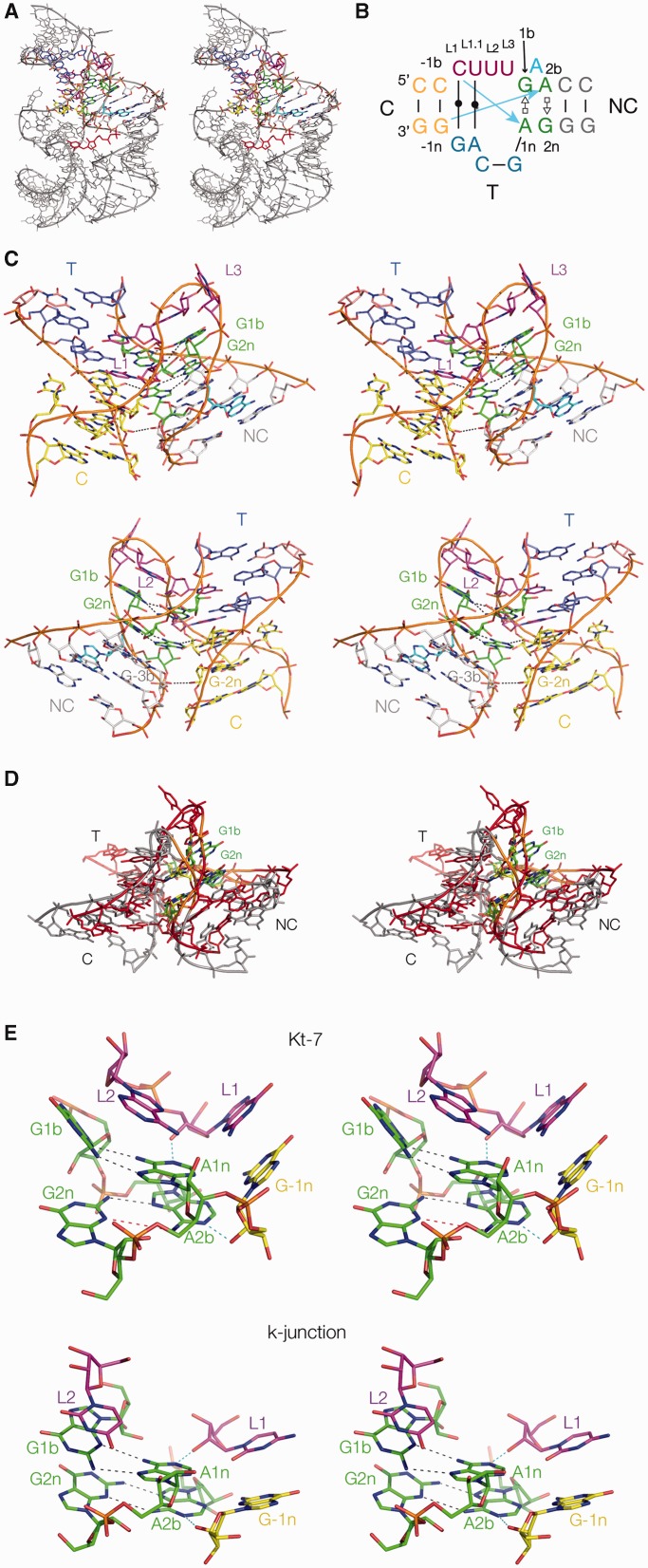
The *A.thaliana* TPP riboswitch and the k-junction. (**A**) The whole riboswitch, with the k-junction highlighted in colour. The structure was determined by Ban and coworkers (20,21), and all molecular graphics images generated from PDB file 3D2G, shown as parallel-eye stereo pairs. The TPP ligand is highlighted in red. (**B**) The sequence and secondary structure of the k-junction. Nucleotides are coloured in the same manner as in the molecular graphics images, and the blue arrows indicate the standard cross-strand hydrogen bonds found in most k-turns. The nucleotides are labelled to follow our standard nomenclature for a simple k-turn (13). (**C**) The TPP riboswitch k-junction. Two views are shown, from the side of the bulged strand (upper) and that of the T helix (lower). The ribbon shows the path of the three strands forming the junction. The T and C helices are coaxial, with continuous base stacking through the point of strand exchange with the NC helix. Note the interaction of the adenine that is interposed between G1b and A2b (coloured cyan) in the major groove of the NC helix, hydrogen bonding with the Hoogsteen edge of A5n, forming a triple-base interaction with the 5b•5n pair. (**D**) Superposition of the k-junction with a standard N1-class k-turn. The k-junction is coloured red with 1b•1n and 2b•2n pairs green, while *H. marismortui* Kt-7 as found in the ribosome is coloured grey with 1b•1n and 2b•2n pairs yellow. (**E**) A comparison of the key interactions at the core of a standard N1-class k-turn (*H. marismortui* Kt-7 in the ribosome, upper) with those found in the k-junction (lower). The two key cross-strand hydrogen bonds are highlighted in cyan. In Kt-7 the A2bN6-G2nN3 distance is 4.7 Å (coloured red), i.e. too long to be hydrogen bonded.

The crystal structure of the TPP riboswitch from the flowering plant *A. thaliana* was solved at 2.25Å resolution by Ban and colleagues (20,21). The overall structure is a three-way helical junction (Figure 2A). Two of the helices include an acute angle that brings them close together, thereby creating a binding pocket for the TPP ligand mediated by S-turns within each helix. These two helices were identified by the structural search as having a global similarity with k-turn structure, prompting a closer examination of the core of the junction. This revealed that the structure shares many of the key features of k-turns, including most of the conserved critical interactions. We subsequently became aware that this similarity was briefly noted by Geary *et al.* (22).

The secondary structure and conformation of the junction are shown in Figure 2B and C. It can be described as a k-turn in which a third helix has been inserted into the non-bulged strand opposite the loop, formally creating a 2HS_2_HS_4_ junction (23). This has the G•A and A•G pairs 3′ to the loop, thus defining the NC helix, while there is a conventionally paired helix 5′ to the loop (thus the C helix). We term the third helix the T helix (third). We have used our standard nomenclature for the nucleotides of k-turns (13), beginning with the G1b and A1n positions, insofar as possible. In the global structure of the junction, the T helix is coaxially aligned with the C helix, making this formally a *L*_ex_ junction (24). Both G•A pairs are *trans* sugar (G)•Hoogsteen (A) pairs just as found in a standard k-turn structure, although the G1b•A1n pair is less buckled than is normally observed in a standard k-turn. Unlike a simple k-turn, an additional adenine is inserted between the G1b and A2b. This is directed away from the helix, re-entering the major groove of the NC helix and making a triple-base interaction with the U5b•A5n base pair via the Hoogsteen edge of A5n.

Upon an initial inspection it seemed that the UUU sequence that precedes the 1b position should be the loop of the k-turn, where the central U is stacked upon the end of the NC helix in the manner of a normal L2 position. Nevertheless, further analysis of the detailed interactions in the core (see below) led us to extend the formal definition of the loop sequence in the 5′ direction to include the preceding C, so that we now define the loop as CUUU, labelled L1, L1.1, L2 and L3 sequentially, whereby L1, L2 and L3 are in positions equivalent to those in the simple k-turn (see Figure 2B). In contrast to a simple k-turn, CL1 and UL1.1 are base paired to the G and A, respectively, from the lower strand (i.e. the non-bulged strand in conventional k-turn), and these base pairs form the central segment of the coaxially stacked C and T helices.

Thus all the global features of a k-turn occur in the TPP junction, and the structure can be superimposed with a standard k-turn such as Kt-7 in the ribosome, using the backbone atoms for nucleotides –1n, L1, 2b, 1n, 2n (Figure 2D) with an RMSD of 1.58Å. We therefore term this structure a k-junction.

### Hydrogen bonding in the core of the *A. thaliana* TPP riboswitch k-junction

The core of the k-junction and key hydrogen bonding interactions are shown in Figure 2E, where they are compared with the standard, simple k-turn Kt-7. The structure of standard k-turns is stabilized by a number of well-conserved hydrogen bonding interactions involving 2-hydroxyl groups (13,15). Of these, two cross-strand hydrogen bonds are accepted by the conserved adenine nucleobases at the 1n and 2b positions, donated by the 2-hydroxyl groups of L1 and –1n, respectively. Atomic mutagenesis whereby the O2′ of L1 from Kt-7 was removed totally prevented its metal ion-induced folding in solution (13). We therefore examined the *A. thaliana* TPP riboswitch k-junction to see if corresponding interactions could be identified.

O2′ of the cytosine designated as L1 (see above) is hydrogen bonded to A1n N1 (O-N distance 2.7Å), and is thus performing the role of L1 in a simple k-turn (Figure 2E). Although the L1 nucleobase participates in a Watson–Crick base pair (unlike the corresponding nucleotide of the simple k-turn), a precedent for this is provided by *H. marismortui* Kt-58. This k-turn has a two-nucleotide AG loop opposed by a single A nucleotide loop; the A is stacked onto the NC helix, thus taking the normal role of L2, and the preceding base paired nucleotide provides the O2′ that donates its proton to A1n (13).

The nucleotide 5′ to L1 is designated –1b, and that to which it is base paired on the opposite strand is –1n. In a simple k-turn the O2′ of –1n donates a hydrogen bond to either N3 or N1 of the conserved A2b nucleobase (15). This –1n O2′ to A2b interaction is found in the k-junction, with an O–N distance 2.9Å (Figure 2E). The acceptor is N1 of A2b, and thus the k-junction is an N1 class k-turn (15). However, there is an interesting difference from the structure of simple N1-class k-turns, where the reorientation of the A2b nucleobases stretches the AN6–GN3 distance to a mean value >4.5Å, so that the pair is normally connected by just a single hydrogen bond between GN6 and AN7 (e.g. see Kt-7 in Figure 2E). In the k-junction this distance is 3.4Å, so borderline hydrogen bonded. The structure of the k-junction provides additional flexibility allowing G2n to follow the rotation of A2b in its reorientation. Perhaps the extra adenine interposed between G1b and A2n allows this to occur. The structure of the k-junction also permits one further stabilizing hydrogen bond to form, between the 2′-hydroxyl groups of C3b and G-2n (O-O distance 3.2Å).

Thus once we have set the component nucleotides in the correct structural reference frame we see that in most respects the *A. thaliana* k-junction is a standard k-turn structure, with all the key hydrogen bonding interactions.

### The *E. coli* TPP riboswitch structure

**Figure 3.**
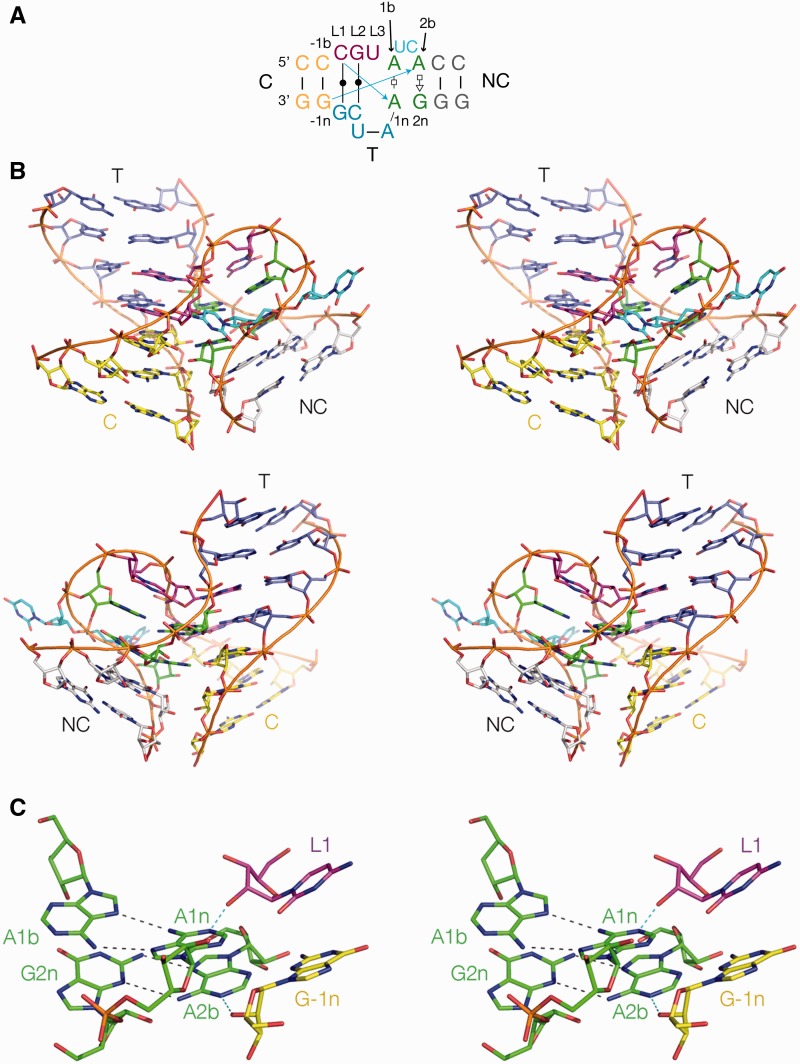
The *E. coli* TPP riboswitch k-junction. The structure was determined by Serganov *et al.* (25), and all molecular graphics images generated from PDB file 2DGI. (**A**) The sequence and secondary structure of the k-junction. Nucleotides are coloured in the same manner as in the molecular graphics images. (**B**) The *E. coli* TPP riboswitch k-junction. Two views are shown, from the bulged strand side (upper), and the T-helix side (lower). (**C**) The core region of the k-junction. The two cross-strand hydrogen bonds are highlighted in cyan.

The observation of the k-junction as the major structural component prompted us to examine carefully the structure of the corresponding TPP riboswitch from *E. coli* solved by Serganov and coworkers (25) at 2.05Å resolution. The global architecture of the bacterial riboswitch is very similar to the plant RNA, being also based on a three-way junction in which the ligand binding site is generated by juxtaposition of two-helical arms of the junction (Figure 3A). We find that the core of the junction is a k-junction that is very similar in all the important aspects to the *A. thaliana* riboswitch, yet with some interesting differences. While the 2b•2n pair is the usual *trans* A(Hoogsteen)•G(sugar) base pair, the 1b nucleotide is an adenine so that the 1b•1n pair is a *trans* A(Hoogsteen)•A(Hoogsteen) base pair that is achieved by placing the A1b in a *syn* conformation. This contrasts with the 2b•2n base pair of *Thelohania solenopsae* Kt-23 for example, which is A(Hoogsteen)•A(sugar) (17). Like the *A. thaliana* k-junction, the 1b and 2b positions are not contiguous, but in this case interrupted by two nucleotides (UC) that do not interact elsewhere within the same molecule. The loop is identified as the CGU sequence (i.e. L1 through L3), but the 5′ C and G are base paired with the opposing CG sequence again making a continuously stacked C-T helix.

The standard k-turn cross-strand hydrogen bonds accepted by the conserved adenine nucleobases are present; CL1 O2′ to A1n (O-N distance 2.5Å) and the G-1n O2′ to A2b (O–N distance 2.9Å) (Figure 3B). The acceptor for the latter is A2b N1, so that the bacterial k-junction is another N1 structure. The AN6–GN3 distance in the 2b•2n pair is 3.4Å, again significantly shorter than simple N1-class k-turns. Thus this k-junction, like that of the *A. thaliana* riboswitch, has all the critical features of a k-turn. Like the *A. thaliana* riboswitch, there is an additional hydrogen bond between G-2n O2′ and A3b O2′ (O–O distance = 2.8Å).

### Effects of nucleotide substitutions within the *E. coli* TPP riboswitch k-junction on folding and ligand binding

We have studied the significance of the k-junction in the folding and function of the *E. coli* TPP riboswitch using two distinct methods. We used gel electrophoresis under non-denaturing conditions to follow changes in the global conformation of the riboswitch RNA. In addition, we have studied the binding of TPP to the RNA using isothermal titration calorimetry (ITC); Ferré D’Amaré *et al.* (19) have previously shown a negative enthalpy of binding of TPP to the *E. coli* riboswitch and thus evolution of heat on binding. We have performed these experiments for the natural riboswitch sequence, and variants with substitutions of nucleotides in the k-junction expected to be important by analogy with their role in k-turns.

**Figure 4.**
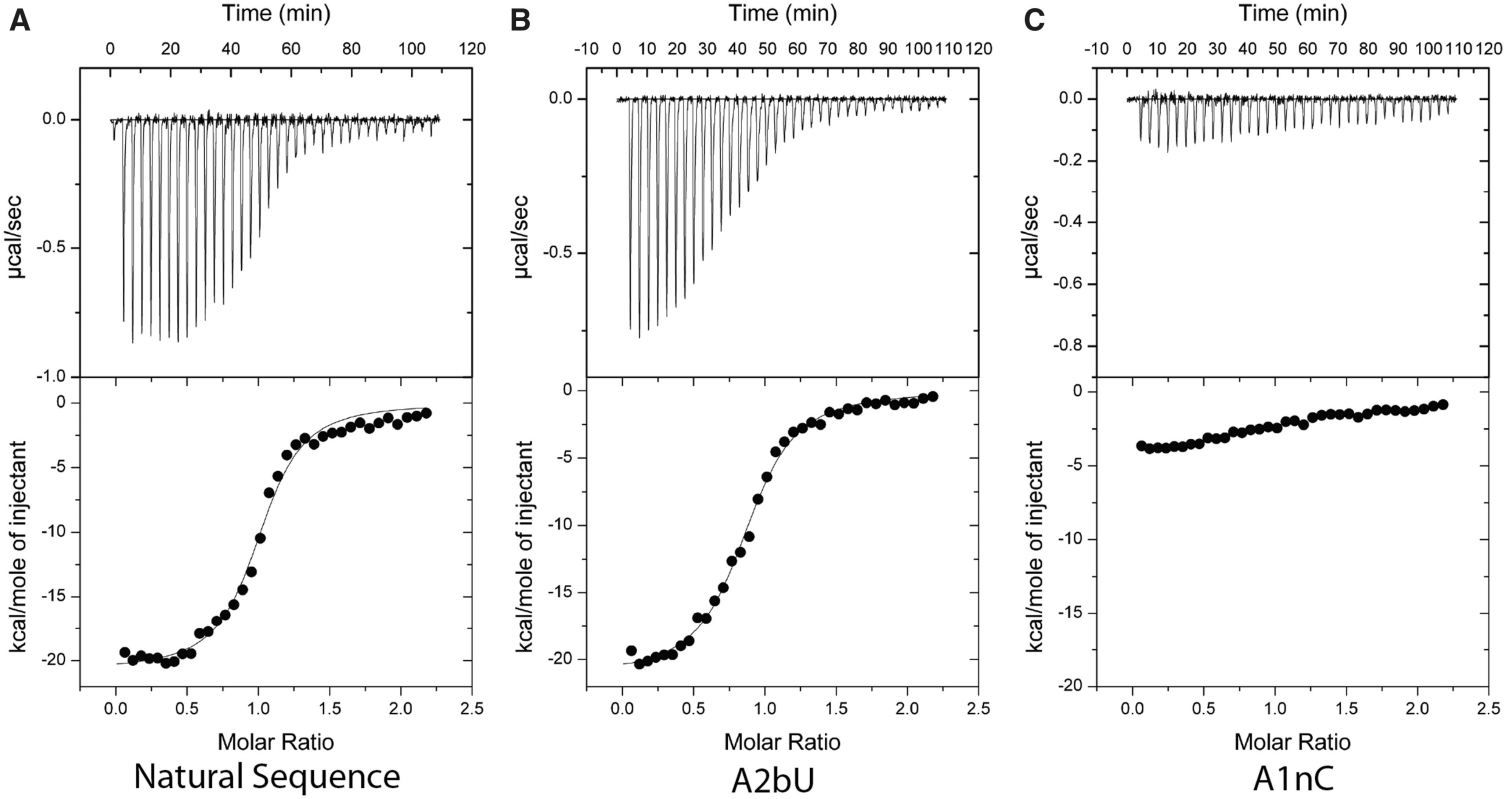
Isothermal titration calorimetric analysis of TPP binding to natural-sequence and variant *E. coli* TPP riboswitch RNA. A solution of SAM was titrated into a SAM-I riboswitch RNA solution, and the heat evolved was measured by ITC as the power required to maintain zero temperature difference with a reference cell at 303.15 K. Integration over time gives the heat required to maintain thermal equilibrium between cells. In each case the upper panel shows the raw data for sequential injections of 8 µl volumes (following an initial injection of 1 µl) of a 150 µM solution of TPP into a 1.4 ml 15 µM RNA solution in 40 mM Hepes (pH 7.5), 100 mM KCl, 10 mM MgCl_2_. This represents the differential of the total heat (i.e. enthalpy Δ*H*° under conditions of constant pressure) for each TPP concentration. The lower panels present the integrated heat data fitted (where possible) to a single-site-binding model. The thermodynamic parameters calculated, together with other variants not shown here, are summarized in Table 1. The ITC analysis was performed for the *E. coli* TPP riboswitch in which the k-junction sequence was modified as (**A**) unmodified k-junction, (**B**) A2bU k-junction and (**C**) A1nC k-junction.

Studied by ITC, the unmodified riboswitch sequence exhibits an exothermic reaction on binding of TPP (Figure 4). The titration data have been fitted, and the resulting thermodynamic parameters tabulated (Table 1), giving a binding affinity of 230 nM. All substitutions within the k-junction resulted in weaker binding, but there was a range of magnitudes. No ligand binding was detected for the A1nC variant, whereas significant binding was observed for substitutions at A2b, although with affinities lowered 2–4 fold. Interestingly an A1bG substitution, which restores the G•A pair at the 1b•1n position of a standard k-turn (and also in the *A. thaliana* TPP riboswitch) leads to a lower affinity of TPP binding by a single order of magnitude.

**Table 1.** Thermodynamic parameters calculated for the *E. coli* TPP riboswitch with and without sequence variations in the k-junction

k-junction	*n*	Δ*H*/kJ.mol^−1^	Δ*S*/J.K^-1^.mol^−1^	Δ*G*/kJ.mol^−1^	*K*_d_/μM
Natural	1.07 ± 0.07	–18.29 ± 0.18	–30 ± 0.63	–9.20 ± 0.06	0.23 ± 0.02
A1bG	0.56 ± 0.01	–17.82 ± 0.45	–33.1 ± 1.50	–7.79 ± 0.06	2.44 ± 0.23
ΔUC	0.82 ± 0.01	–8.72 ± 0.12	1.24 ± 0.47	–9.09 ± 0.08	0.28 ± 0.04
A1nU	0.93 ± 0.04	–11.81 ± 0.79	–14.3 ± 2.63	–7.48 ± 0.11	4.07 ± 0.73
A2bC	0.91 ± 0.02	–21.85 ± 0.47	–44.3 ± 1.57	–8.42 ± 0.08	0.85 ± 0.11
A2bU	0.88 ± 0.01	–21.18 ± 0.24	–41.2 ± 0.81	–8.69 ± 0.05	0.55 ± 0.04

Note that the A1nC variant is not included, because no binding was detectable with the data obtained; *n* is the stoichiometry of the interaction.

**Figure 5.**
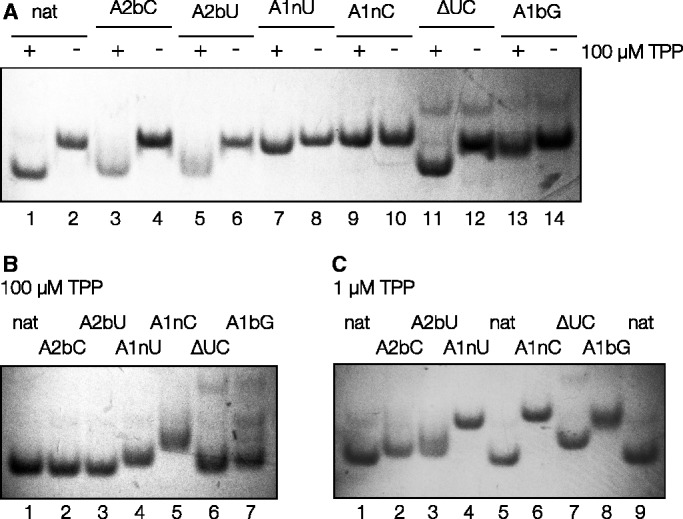
Analysis of the global structure of *E. coli* TPP riboswitch RNA as a function of the presence of TPP using gel electrophoresis. This was performed for natural sequence riboswitch RNA, and variants with modifications in the k-junction. RNA samples were electrophoresed in 10% polyacrylamide gels in the presence of 25 mM Tris, 192 mM glycine (pH 8.3), 1 mM Mg^2+^ ions. RNA was visualized by UV shadowing. (**A**) RNA was incubated with (+) or without (–) the addition of 100 µM TPP before application to the gel and electrophoresis for the natural-sequence RNA and the indicated variants. Note the marked increase in mobility for the natural-sequence riboswitch RNA preincubated in the presence of TPP. Tracks: 1 and 2, unmodified natural-sequence riboswitch (nat); 3 and 4, riboswitch with A2bC substitution; 5 and 6, A2bU; 7 and 8, A1nU; 9 and 10, A1nC; 11 and 12, riboswitch with a deletion of the AU between the 1b and 2b positions; 13 and 14, A1bG (creating a G•U pair at the 1b•1n position). For odd numbered tracks the RNA was preincubated with 100 µM TPP, for even numbered tracks no TPP was present. (**B** and **C**) The same RNA species were applied to a gel without preincubation. In this case, the electrophoresis running buffer contained (B) 100 µM TPP or (C) 1 µM TPP in addition to the indicated components. Tracks: (B) 1, unmodified natural-sequence riboswitch; 2, A2bC substitution; 3, A2bU; 4, A1nU; 5, A1nC; 6, riboswitch with a deletion of the AU between the 1b and 2b positions; 7, A1bG; (C) 1, unmodified natural-sequence riboswitch; 2, A2bC; 3, A2bU; 4, A1nU; 5, unmodified riboswitch; 6, A1nC; 7, riboswitch with a deletion of the AU between the 1b and 2b positions; 8, A1bG; 9, unmodified riboswitch.

The global conformation of the riboswitch RNA was studied by gel electrophoresis that was performed in two alternative ways (Figure 5). In the first the RNA was incubated with or without 100 µM TPP and then electrophoresed in polyacrylamide in a buffer without added TPP (Figure 5A). In the second the electrophoresis was performed with TPP continuously present in the running buffer (Figure 5B and C). Two different concentrations of TPP were used, either 100 µM (a saturating ligand concentration, Figure 5B) or 1 µM (close to the K_d_ for the natural-sequence riboswitch, Figure 5C). In the buffer for all gels, 1 mM Mg^2+^ ions were present. In agreement with previous observations (19), the natural-sequence riboswitch RNA forms a tight band in the gel that migrates significantly faster when TPP is present (Figure 5A, tracks 1 and 2). The difference in mobility with and without addition of TPP reflects a significant change in the global conformation, revealing that binding of the TPP is required to achieve the fully folded conformation of the riboswitch. Moreover it shows that ligand release and consequent unfolding occur slowly compared to the timescale of the electrophoresis.

In contrast, the A1nC variant exhibits no increase in mobility on addition of TPP (Figure 5A, tracks 9 and 10), and was apparently unfolded even in a high concentration of the ligand (Figure 5B, track 5). Clearly this substitution leads to a failure to undergo TPP-induced folding, consistent with the absence of binding detectable by calorimetry. A1nU substitution also has a major effect, although not as great as A1nC. An intermediate mobility in polyacrylamide is observed (e.g. Figure 5B, track 4), likely corresponding to an incomplete population of the folded form, and an affinity of binding that is lowered 17-fold. The A1bG variant exhibits partial folding, and the difference from the natural sequence is particularly clear in the presence of 1 µM TPP (Figure 5C, track 8). With a G•A pair at the 1b•1n position the riboswitch evidently releases bound TPP more rapidly, consistent with the lowered affinity. The reason for this is unclear at the present time. The effects of substitutions at the 2n position are smaller. Turning to the 2b position, with no TPP added to the running buffer (Figure 5A, tracks 3 and 5) or with 1 µM TPP in the buffer (Figure 5C, tracks 2 and 3), the bands of the A2bU and A2bC variants were less tight, and smeared towards lower mobility, suggesting that the ligand was slowly released from the RNA during the electrophoresis process. ITC also revealed a slightly lowered affinity of binding. However, electrophoretic migration of the 2 b variants in the presence of 100 µM TPP was almost the same as for the natural riboswitch (Figure 5B, tracks 2 and 3), indicating that a high ligand concentration can force the population into the folded form. Deletion of the UC bulge (ΔUC) between the 1b and 2b positions had the smallest effect on folding and binding. An intermediate migration was observed in the presence of 1 µM TPP (Figure 5C, track 7), but at the saturating concentration of ligand (Figure 5B track 6) the migration was close to that of the unmodified riboswitch. The affinity of binding was lowered by less than a factor of 2.

In summary, the substitutions reveal the importance of the k-junction to the folding of the riboswitch and its ability to bind TPP ligand, and sensitivity to substitution in the order 1n > 2b > UC bulge as expected by analogy with the simple k-turn.

## DISCUSSION

The identification of the k-junction in the TPP riboswitches shows that the k-turn is an adaptable motif that can be accommodated within the framework of more complex structural entities. In such guises the structure can go undiscovered within larger RNA species. Yet the k-junction shows that the essential elements of k-turn structure provide a good platform to construct three-way helical junctions, and this in turn can be used to generate a ligand-binding cavity between two arms. Indeed, this is critical to the function of the TPP riboswitch, modulating the overall stability so that it exhibits the required differential stability with and without bound ligand.

The k-junction fits perfectly into the context of a three-helix junction of the *L*_ex_ type (24), a member of the family B of Lescoute and Westhof (26). The loop of the k-turn forms the exiting segment, of which two nucleotides are each base paired to maintain perfect stacking through strand exchange region. In this way, the T and C helices are continuously coaxially stacked, while the NC helix forms the unstacked helix. All the standard interactions of the simple k-turn are present within the junction structure. The k-junction is stabilized by a further cross-strand hydrogen bond between G-2n O2′ and A3b O2′; this interaction is also found in a subset of simple k-turns such as Kt-7 in the ribosomal context (13). Thus there is a network of hydrogen bonds lacing up the interface between the NC and C helices of the k-junction. We have shown that substitution of any of the key nucleotides contributing to k-turn structure disrupts the folding and weakens ligand binding to one extent or another, of which the most important is the 1b•1n interaction as shown for simple k-turns (e.g. as shown in the SAM-I riboswitch (8)). The k-turn structure is accommodated within the junction with minimal disruption, aside from the increased planarity of the 1b•1n pair.

**Figure 6.**
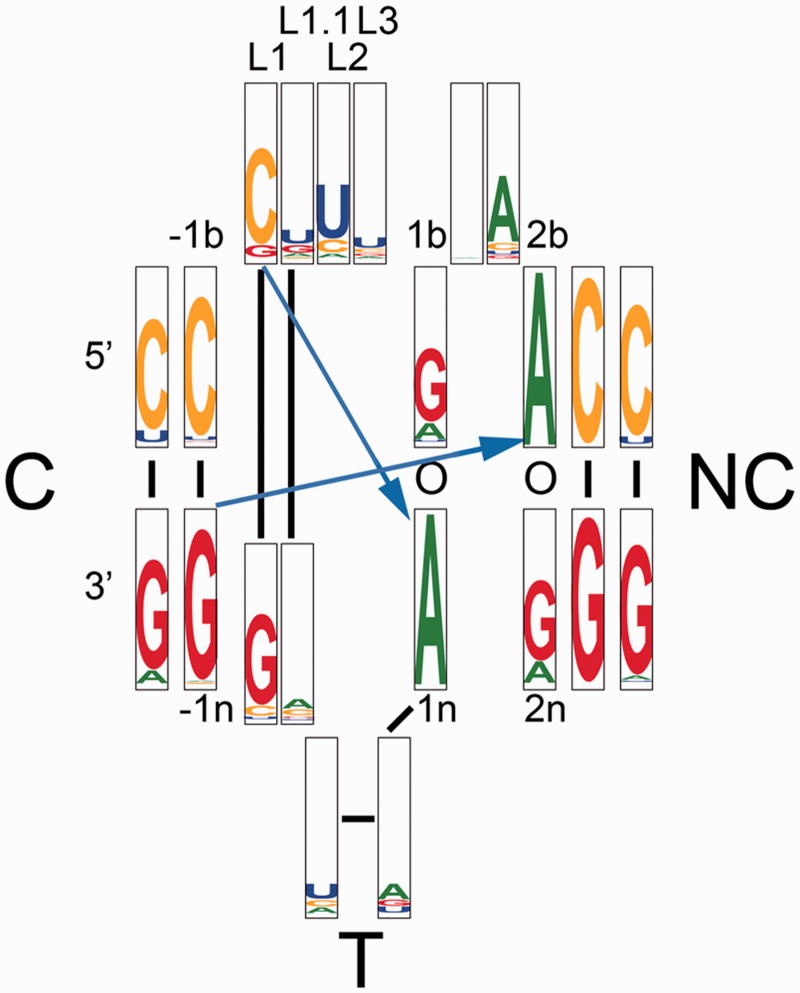
Distribution of sequences in the TPP riboswitches. Of available sequences, 11 056 were analyzed and presented as a positional entropy plot (27) of frequency of occurrence. A sequence logo representing the nucleotide conservation as well as information content at each position in a structure-based alignment of sequences. For each position, the height of each nucleotide symbol represents its frequency at that position, while the overall height represents the information content at that position. The sequences were taken from the Rfam database (18), accession number RF00059. The k-junction region was manually aligned against the crystal structures of the TPP riboswitches using Jalview (28).

How general is the k-junction in RNA structure? Analysis of 11 056 TPP riboswitch sequences suggests that the essential features of the k-junction is preserved in this large sample (Figure 6). The most conserved nucleotides are the critical adenines at the 1n and 2b positions that accept the cross-strand hydrogen bonds, at 99.52 and 99.96%, respectively. The 1b•1n pair is G•A in 75.76% of sequences, but replaced by A•A (as it is in the *E. coli* sequence) in 16.89%. The analysis also shows that 21.53% of sequences have an A•A pair at the 2b•2n position. The 3b•3n CG base pair is strongly conserved at 99.10%. An analysis of the full riboswitch is presented in Supplementary Figure S1. This reveals that the most conserved nucleotides are those contributing to the ligand binding pocket, and those of the k-junction.

The k-junction is not restricted to the TPP riboswitches, and we have found further examples in other RNA species. A k-junction is found the large ribosomal subunit of *H. marismortui* in the J4,5 region. Interestingly, this was previously identified as a k-turn (1), but close inspection shows that it is a k-junction sharing many of the same features as those of the two riboswitches. Thus it is another three-way junction, formed by a k-turn that is elaborated by a branchpoint in the T helix (Supplementary Figure S2). There is a standard G1b•A1n base pair, but a *trans* A2b(Hoogsteen)•A2n(Hoogsteen) pair. In contrast to the riboswitch k-junctions there are no nucleotides separating G1b and A2b, and the G1b•A1n pair is significantly buckled. The loop is clearly identified as the preceding GUA sequence and designated L1 through L3 with their usual structural functions. The C helix begins with the uridine immediately 5′ to GL1. The structure of the non-bulged strand of the NC helix does not map linearly onto the sequence. In terms of the sequence the nucleotides run 5′ to 3′ as G3n A1n A2n U; the final uridine is base paired with AL2, requiring an S-shaped turn to form in the backbone. A similarly ‘out of sequence’ non-bulged strand is found in the NC helix of the complex k-turn Kt-11 of *T.thermophilus*, and we may therefore regard this as a complex k-junction. As previously, we designate the nucleotides in terms of their role in the structure, so that A1n is paired with G1b for example. With the components of the k-junction assigned we find that all the standard hydrogen bonding interactions are present. L1O2′ donates a proton to A1n N1 (O–N distance = 2.7Å), and A-1n O2′ to A2b N1 (O–N distance = 2.7Å). Thus this is another N1 class structure, and the A2b N6 to A2n N7 distance is 3.5Å. Like the simple k-turns, the L3 O2′ to L1/L2 phosphate *pro*S O is found in this structure (O–O distance = 2.7Å), and like the riboswitch k-junctions one further hydrogen bond forms between G-2n O2′ and A3b O2′ (O–O distance = 3.3Å).

The importance of the k-junction motif is further underlined by finding a second example in the *H.marismortui* 50S ribosomal subunit, at J94/99 (Supplementary Figure S3). Although the T helix is only partially defined by the available structure this is plainly a three-helix junction that is similar to those of the TPP riboswitch. The three-base GCC loop can be defined according to function as L1 (stacked onto the C helix), L1.1 and L2 (stacked onto the NC helix). The NC helix begins with a standard *trans* G(sugar)•A(Hoogsteen) pair at the 1b•1n position. Once again this is almost planar, in contrast to those of the simple k-turns. A1n N1 accepts the standard hydrogen bond from L1 O2′ (O–N distance = 2.7 Å). The 2b•2n position is a *trans* A(Hoogsteen)•A(sugar) pair connected by a single hydrogen bond. A2b N1 accepts a hydrogen bond from –1n O2′ (O–N distance = 2.9 Å). Thus both the standard cross-strand hydrogen bonds are present, and once again this is an N1-class structure.

Our analysis shows that the basic elements of k-turn structure are well suited to accommodate a three-way helical junction, preserving all the key features and interactions of the k-turn. This has been extensively exploited in the TPP riboswitches, but clearly occurs more widely as demonstrated by the two examples found in the *H. marismortui* ribosome. Given that the k-junctions are not immediately easy to recognize, and indeed have been overlooked in a number of structures, it is likely that they are relatively widespread and that more examples remain to be discovered.

## SUPPLEMENTARY DATA

Supplementary Data are available at NAR Online.

Supplementary Data
