# Supplementary material for: The k-junction motif in RNA structure
Source: Nucleic Acids Res. 2014 Feb 14;42(8):5322–31. doi: 10.1093/nar/gku144 (PMC4005666; doi:10.1093/nar/gku144)
Supplement: Supplementary Data [file supp_42_8_5322__index.html]

The k-junction motif in RNA structure — The k-junction motif in RNA structure — Supplementary Data 

# The k-junction motif in RNA structure

## Supplementary Data

files

**Files in this Data Supplement:**

- Supplementary Data - zip file
