# Supplementary material for: The k-junction motif in RNA structure
Source: Nucleic Acids Res. 2014 Feb 14;42(8):5322–31. doi: 10.1093/nar/gku144 (PMC4005666; doi:10.1093/nar/gku144)
Supplement: Supplementary Data [file supp_gku144_suppl_data.zip › nar-03383-r-2013-File014.pdf]

The k-junction motif in RNA structure

Jia Wang, Peter Daldrop, Lin Huang and David M. J. Lilley

Supplementary Information

S1. Supplementary Figures

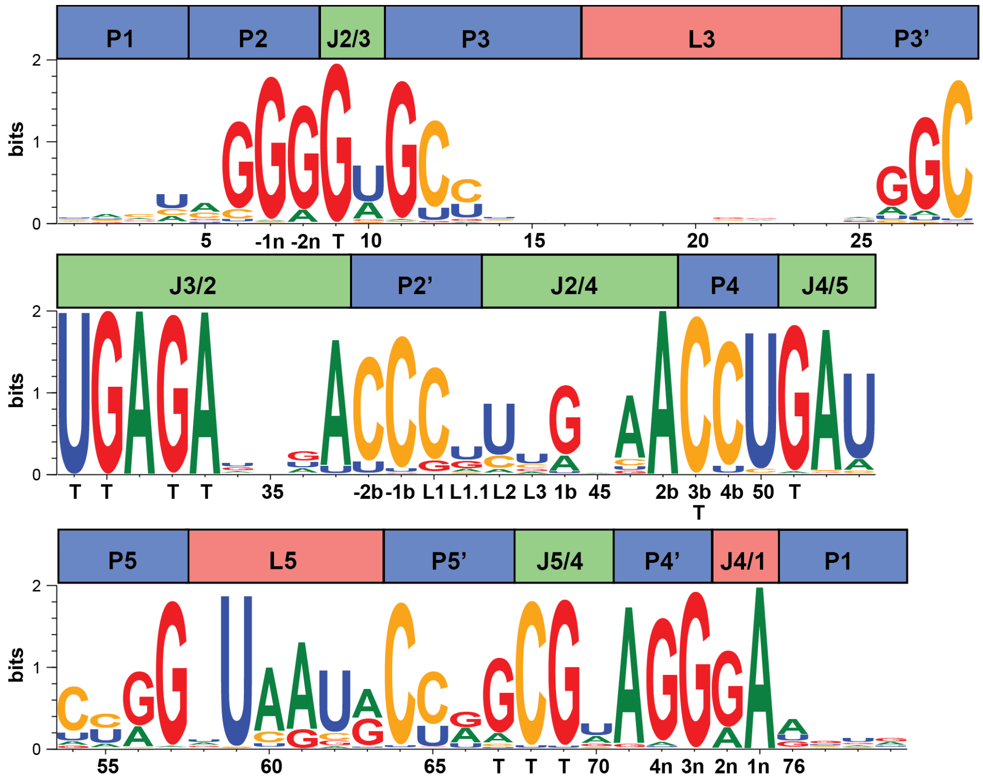

Figure S1. Positional entropy plot for the complete TPP riboswitch A sequence logo representing the nucleotide conservation as well as information content at each position in a structure-based alignment of 11,056 sequences. For each position, the height of each nucleotide symbol represents its frequency at that position, while the overall height represents the information content at that position. The nucleotides of the k-junction region in the sequence are labelled as their position in a standard k-turn. T indicates TPP binding sites. Note that the most conserved nucleotides are those contributing to the ligand binding pocket, and those of the k-junction.

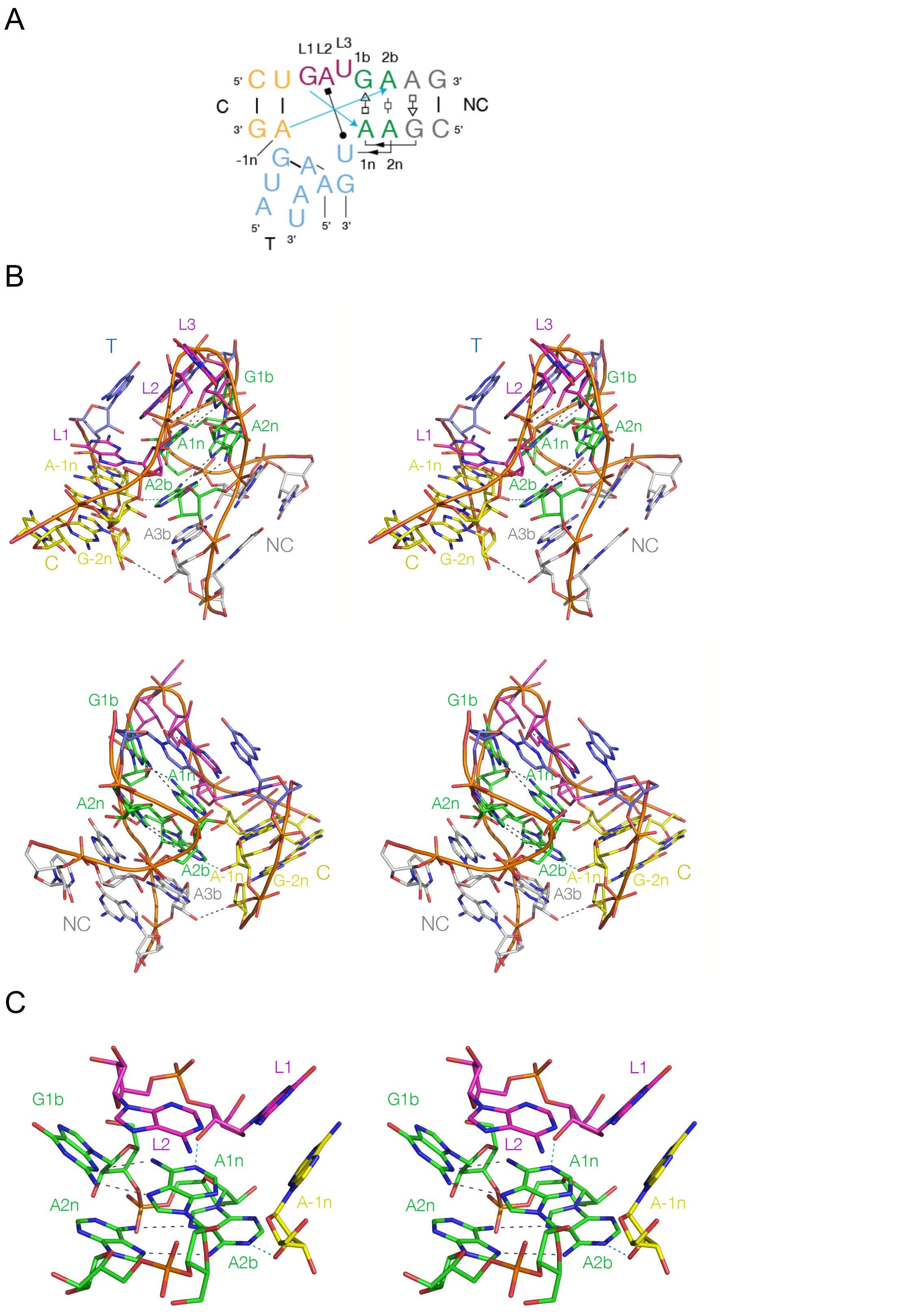

Figure S2. The structure of the J4/5 k-junction of the *H. marismortui* 50S ribosomal subunit (PDB ID 3CC2).

A. The sequence and secondary structure of the J4/5 k-junction. Note the inversion of the non-bulged strand so that the sequence reads 5' G A1n A2n U 3' passing into the T helix.
B. Parallel-eye stereoscopic views of the k-junction from the side of the bulged strand (upper) and that of the T helix (lower). The S-turn in the ribose-phosphate backbone trajectory of the non-bulged strand in the NC helix inverts A1n and A2n. Note the additional cross-strand hydrogen bond between the 2'-hydroxyl groups of G-2n and A3b.
C. The core region of the k-junction. The two key cross-strand hydrogen bonds are highlighted in cyan.

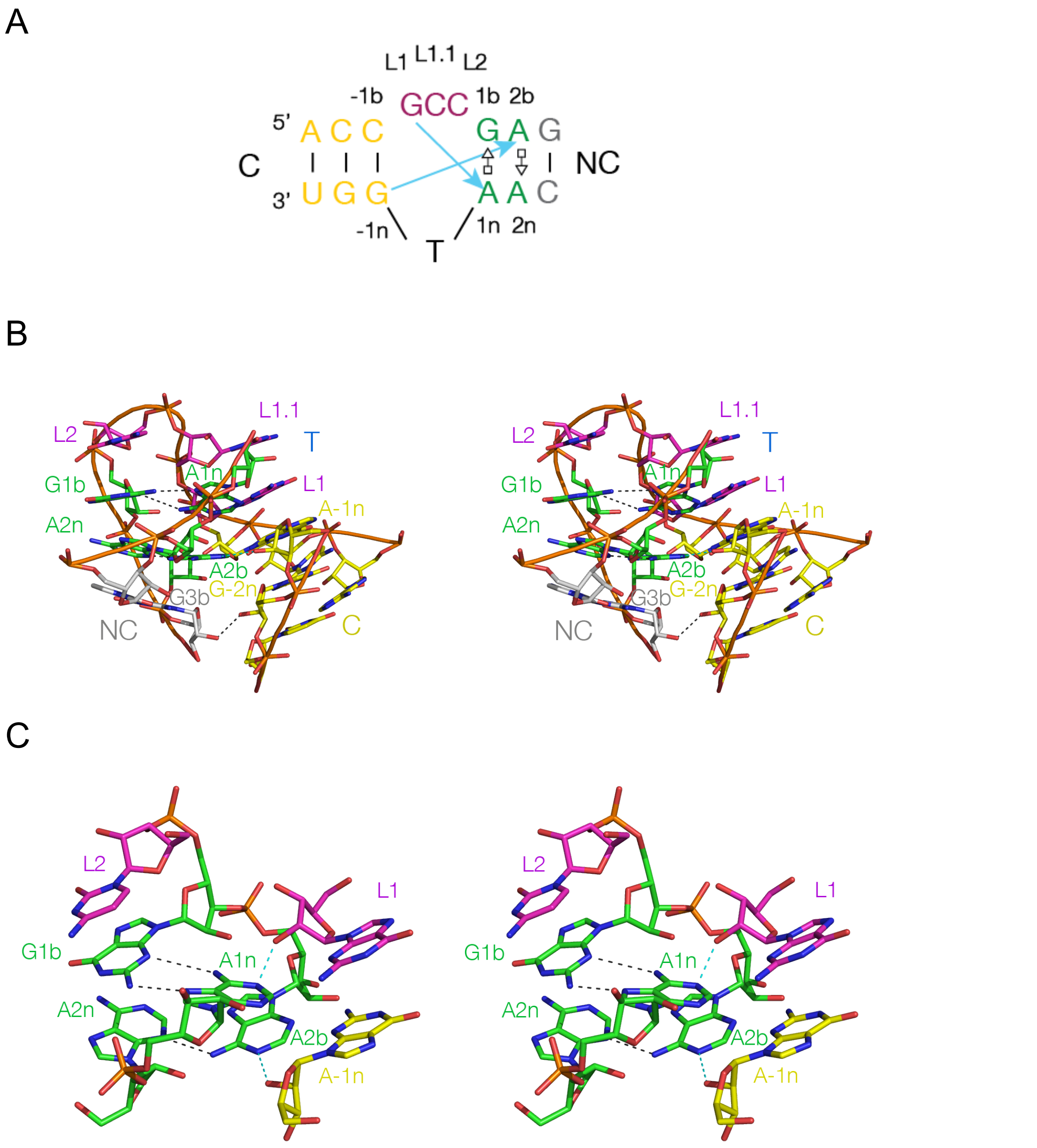

Figure S3. The structure of the J94/99 k-junction of the *H. marismortui* 50S ribosomal subunit (PDB ID 3CC2).

A. The sequence and secondary structure of the J94/99 k-junction.

B. Parallel-eye stereoscopic view of the k-junction from the side of the T helix. Note the additional cross-strand hydrogen bond between the 2'-hydroxyl groups of G-2n and G3b.

C. The core region of the k-junction. The two key cross-strand hydrogen bonds are highlighted in cyan.

S2. Description of the scripts used to search for k-turn-related structures in RNA database

The procedure to search for k-turn-like motifs using computer-guided visual analysis comprises four separate stages :

1. Preparation of a list of pdb-files to be screened
2. Preparation of a motif-definition file to define the search pattern
3. Screening the pdb files
4. Visual analysis of the results

General

Files names are usually hardcoded. User must adjust to match the file locations on his/her computer. Also all python extensions given at the top of the scripts must be present, especially BioPython. Furthermore we strongly advise testing the procedure using a small set of pdb files (e.g. 5-10) before running this over the whole pdb.

1. Preparation of a list of pdb files to be screened

Any RNA-containing pdb file can be studied. All pdb-files to be scanned need to be placed into a folder that contains only these files. Download the lists of pdb entries with mixed polymers and mixed polymers without RNA from www.pdb.org as textfiles and use strucloader1 to download all RNA-containing pdb files into a folder specified in the scripts. The RNAextractor script then isolates the RNA portion of these files and generates a folder containing the resulting pdb files containing only the RNA coordinates. Next the RNA files need to be analysed to find the helix segments by using batchSegmenter which in turn requires the code from auxfuncs and basep. This file then creates a single file in an output directory for a single RNA species.

2. Preparation of a motif-definition file to define the search pattern

This is done by executing the motifdef script on a pdb file containing exactly two helix segments. Select 2 segments by loading the segment file and the corresponding RNA pdb file into a molecular viewer and find the two segments to define a given motif. Then use these two segments as input for the script.

The following k-turns were used to define the k-turn global geometry:

1. 4B5R Kt-7 in the SAM-I riboswitch
2. 3CC2 Kt-7 in the *H. marismortui* ribosome
3. 3CC2 Kt-58 in the *H. marismortui* ribosome
4. 3CC2 Kt-42 in the *H. marismortui* ribosome

5. 3CC2 Kt-38 in the *H. marismortui* ribosome
6. 1E7K U4 snRNA k-turn
For each k-turn, coordinates for all pseudoatoms of an NC helix segment were expressed in coordinates defined by a C-helix segment. These relative coordinates were stored in a motif definition file.

A file containing more than one motif can be constructed by concatenating several output flies from motifdef, but edit the names of the motifs in the files to avoid confusion.

3. Screening the pdb files

The helix-segment files of the structures and the motif-definition file now serve as input for the structural screening process. The batchScreener1 file organizes the screen by calling the screener1 script for each segment file.
For each k-turn, coordinates for all pseudoatoms of an NC helix segment are expressed in coordinates defined by a C-helix segment. These relative coordinates are stored in a motif definition file.
To identify possible motifs in a given file of RNA segments, each segment is checked for candidate partner segments. For each segment, candidate partner segments are identified within a cut-off from the ideal position. Further, the cosine between the z axis of the candidate segment and the ideal z axis from the motif definition is calculated. This is also calculated for the y axis. A motif is scored positive if the distance between origin and ideal origin is less than the cut-off, and if the product of the z and y axis cosines is greater than a set threshold.
Accepted motifs are written into a pdb file. The origin of the C-helix segment is represented by a pseudo C-atom and the NC-helix segment by a pseudo N-atom.
Furthermore the motif is scored as follows:
distscore = (d_max-d)/d_max,
y_score = cos(y, ideal_y)
z_score = cos(z, ideal_z),
Total_score = distscore*y_score*z_score.
Scores are written as a remark into the output pdb file.

4. Visual analysis of the results

The results can now be analysed by viewing the resulting pdb files together with the corresponding pdb files. To facilitate this, the analyser script can be used to compile a spreadsheet in csv format listing all results with their pdb code, structure number and score. This script needs the results folder and the folder with the original pdb files as

input. It outputs a single csv spreadsheet. Script resultsorter extracts the non-ribosomal structures from the total.

Script (.py)	Function
strucloader1	generates a list of all pdb files containing RNA given the lists of pdb-files containing RNA only and mixed polymers
RNAextractor	takes a folder containing only pdb files and writes all complete RNA nucleotides for each pdb-file into an output file pdb file
basep	contains the class and methods to identify the base pairs
auxfuncs	code file containing various bits of codes that are shared between the scripts
batchSegmenter	This file generates the list of helix segments used by the screener1/batchScreener1 to identify the motif candidates.
motifdef	generates the search pattern from a file containing helix segments
batchScreener1	Calls screener1 for all files in a list of files
screener1	performs the actual screen for a pdb file containing helix elements. Is called by batchScreener1 which manages screening of a list of structures
analyser	this file looks at all results files and generates the table with all results which can be opened in Excel
resultsorter	separates ribosomal structures from non-ribosomal

S3. Tabulation of PDB files referred to in the text

Species	RNA molecule	k-turn	PDB	Chain ID	2b Res. No	1n Res. No	Reference
H. marismortui	23S	Kt-7	3CC2	0	98	80	(a)
T. thermophilus	16S	Kt-23	2WH1	A	704	687	(b)
T. solenopsae	16S	Kt-23	4AOB	A	36	20	(c)
T. thermophilus	16S	Kt-11	2WH1	A	282	246	(b)
H. marismortui	23S	Kt-15	3CC2	0	265	247	(a)
H. marismortui	23S	Kt-58	3CC2	0	1606	1590	(a)
H. sapiens	U4 snRNA	U4	1E7K	C	33	44	(d)
A. thaliana	TPP riboswitch	k-junction	3D2G	A	44	72	(e)
E. coli	TPP riboswitch	k-junction	2GDI	A	56	84	(f)
H. marismortui	23S	J4/5	3CC2	0	48	113	(a)
H. marismortui	23S	J94/99	3CC2	0	2827	2914	(a)

Table S1. The PDB files for all the k-turns and k-junctions discussed in this paper. For each we list the PDB ID, the chain ID and the nucleotide numbers of the 2b and 1n positions. The references for these structures are :

(a) Blaha, G., Gurel, G., Schroeder, S.J., Moore, P.B. and Steitz, T.A. (2008) Mutations outside the anisomycin-binding site can make ribosomes drug-resistant. *J Molec. Biol.*, **379**, 505-519.

(b) Weixlbaumer, A., Jin, H., Neubauer, C., Voorhees, R.M., Petry, S., Kelley, A.C. and Ramakrishnan, V. (2008) Insights into translational termination from the structure of RF2 bound to the ribosome. *Science*, **322**, 953-536.

(c) Schroeder, K.T., Daldrop, P., McPhee, S.A. and Lilley, D.M.J. (2012) Structure and folding of a rare, natural kink turn in RNA with an A•A pair at the 2b•2n position. *RNA*, **18**, 1257-1266.

(d) Vidovic, I., Nottrott, S., Hartmuth, K., Luhrmann, R. and Ficner, R. (2000) Crystal structure of the spliceosomal 15.5 kD protein bound to a U4 snRNA fragment. *Molec. Cell*, **6**, 1331-1342.

(e) Thore, S., Frick, C. and Ban, N. (2008) Structural basis of thiamine pyrophosphate analogues binding to the eukaryotic riboswitch. *J. Amer. Chem. Soc.*, **130**, 8116-8117.

(f) Serganov, A., Polonskaia, A., Phan, A.T., Breaker, R.R. and Patel, D.J. (2006) Structural basis for gene regulation by a thiamine pyrophosphate-sensing riboswitch. *Nature*, **441**, 1167-1171.

These structures can be viewed at : http://www.dundee.ac.uk/biocentre/nasg/kturn/index.php see Schroeder, K.T., McPhee, S.A., Ouellet, J. and Lilley, D.M.J. (2010) A structural database for k-turn motifs in RNA. *RNA*, **16**, 1463-1468.

S4. Files available for download

1. Python scripts used in structural screening of database

The 10 Python scripts required for the analysis are placed in a folder in compressed form called Wang_Scripts.zip .

2. Tabulation of structures identified by structural screen.

The results of the database screening for k-turn-like structures are presented in a spreadsheet called Wang_Hits.xlsx . The first tab presents all the results, while the second excludes those of ribosomal origin.
